# WYBQ-4: a New Bactericidal Agent against Methicillin-Resistant Staphylococcus aureus

**DOI:** 10.1128/spectrum.00547-22

**Published:** 2022-09-13

**Authors:** Shuhan Guan, Hangqian Yu, Hua Xiang, Li Wang, Jingyu Liu, Anfang Wu, Jianze Zheng, Hongbo Dong, Lin Wang, Dacheng Wang

**Affiliations:** a College of Animal Science, Jilin University, Changchun, China; b State Key Laboratory for Zoonotic Diseases, Key Laboratory for Zoonosis Research of the Ministry of Education, Institute of Zoonosis, and College of Veterinary Medicine, Jilin University, Changchun, China; c College of Animal Medicine, Jilin Agricultural University, Changchun, China; d Changchun University of Chinese Medicine, Changchun, China; e School of Pharmacy, Chengdu University, Chengdu, China; University of Calgary

**Keywords:** methicillin-resistant *Staphylococcus aureus*, WYBQ-4, PBP2a, bactericidal agent

## Abstract

Methicillin-resistant Staphylococcus aureus (MRSA) is a multidrug-resistant pathogen that currently poses a serious threat to global health. Novel antimicrobial agents against MRSA are urgently being developed. In this study, we investigated WYBQ-4, which is an effective antibacterial agent with potent bactericidal activity and bactericidal efficiency against MRSA USA300 and clinical isolate strains. In addition, WYBQ-4 exhibited low cytotoxicity without hemolytic activity according to a safety evaluation. Importantly, WYBQ-4 showed potent *in vivo* efficacy in an MRSA-induced mouse pneumonia model, systemic infection model, and intramuscular infection model. The efficacy of this new cephalosporin against MRSA was associated with a high affinity for penicillin-binding proteins (PBP1, PBP2, PBP3, PBP4, PBP2a) evaluated in a competition assay using bocillin as a reporter. In conclusion, WYBQ-4 has a significant bactericidal effect *in vitro* and *in vivo*, indicating that it is a promising compound to control MRSA infection.

**IMPORTANCE** Antibiotic resistance is spreading faster than the introduction of new compounds into clinical practice, causing a public health crisis. Novel antimicrobial agents against MRSA are urgently being developed. In this study, we investigated WYBQ-4, which is an effective antibacterial agent with potent bacteriostatic activity and bactericidal efficiency against MRSA USA300 and clinical isolate strains. WYBQ-4 showed potent *in vivo* efficacy in MRSA-induced mouse models. Subsequently, we further revealed its antibacterial mechanism. In conclusion, WYBQ-4 has a significant bactericidal effect *in vitro* and *in vivo*, indicating that it is a promising compound to control MRSA infection.

## INTRODUCTION

The evolution, accumulation, and spread of methicillin-resistant Staphylococcus aureus (MRSA) pose a significant public health risk to humans. MRSA is a major source of infection in hospitals and clinics, ranging from soft tissue infections to bacteremia, endocarditis, pneumonia, and osteomyelitis, and is a leading cause of morbidity and mortality worldwide (1, 2). MRSA was listed as a high-priority pathogen in the World Health Organization’s report “List of Priority Pathogens for New Antibiotic Development” as early as 2017, which underscores the urgent need for the development of novel antimicrobials (3).

Currently, antibiotics play a predominant role in the prevention and treatment of diseases caused by MRSA. However, the emergence and rapid spread of MRSA has limited the use of β-lactam antibiotics. The development and application of anti-MRSA drugs in just a few years have led to the emergence of resistant strains and the current status of reduced susceptibility to glycopeptides, daptomycin, and linezolid (4, 5). In addition, diseases caused by MRSA have high morbidity and mortality, and their treatments are extremely difficult. At present, as the pace of exploitation of new antibiotics is still slow, highly effective drugs against MRSA infection are urgently needed (6).

**FIG 1:**
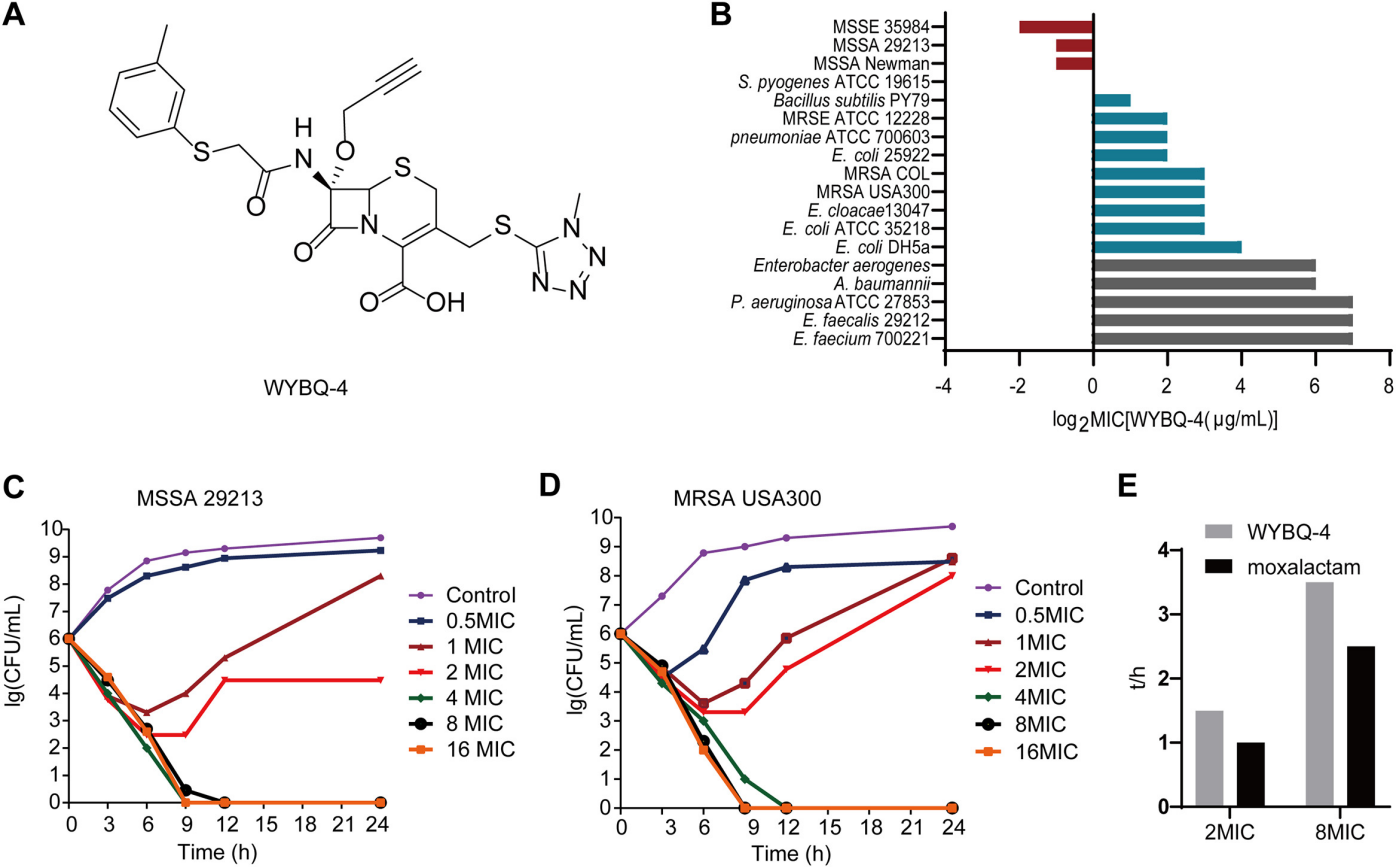
The bactericidal activity of WYBQ-4 *in vitro*. (A) Chemical structure of WYBQ-4. (B) The MICs of WYBQ-4 against Gram-negative and Gram-positive bacteria. (C) Time-kill curve of MSSA 29213 in the presence of WYBQ-4. (D) Time-kill curve of MRSA USA300 in the presence of WYBQ-4. (E) Postantibiotic effects of WYBQ-4 and moxalactam on MRSA.

Novel cephalosporins have attracted increased attention. Optimization and modification of cephalosporin antibiotics have significant value for exploring novel anti-MRSA drugs (7). WYBQ-4 (C_22_H_22_N_6_O_5_S_3_; Fig. 1A) is a new agent that is further optimized based on the structure of cephamycin C. It has a trans-methoxy at the 7α-position on its β-lactam ring, which can effectively form a steric hindrance, and thus has a relatively strong stability to β-lactamases (8).

Subsequently, the anti-MRSA activity and mechanism of the cephalosporin compound WYBQ-4 were investigated. The results showed that WYBQ-4 has significant antibacterial activity against MRSA *in vitro* and that WYBQ-4 can protect mice from a lethal S. aureus challenge *in vivo*. Furthermore, using the bocillin assay, we confirmed that WYBQ-4 could bind to penicillin-binding proteins (PBPs; PBP1, PBP2, PBP3, PBP4, PBP2a) and inhibit S. aureus cell wall synthesis by inhibiting transpeptide action, which is similar to ceftobiprole (9). Collectively, WYBQ-4 is an excellent candidate for developing novel cephalosporin against multidrug-resistant S. aureus infections, which can help to alleviate the current problem of S. aureus resistance due to the misuse of broad-spectrum antibacterial drugs.

## RESULTS

### Significant antibacterial activity of WYBQ-4 against S. aureus and its clinical strains.

The spectrum of activity of WYBQ-4 was determined by measuring its MIC against a panel of Gram-positive bacteria and Gram-negative bacteria. As shown in Fig. 1B, WYBQ-4 had strong antibacterial activity against methicillin-susceptible Staphylococcus aureus (MSSA) 29213 (MIC, 0.5 μg/mL), MRSA USA300 (MIC, 8 μg/mL), methicillin-resistant Staphylococcus epidermidis (MRSE) 12228 (MIC, 4 μg/mL), and methicillin-susceptible Staphylococcus epidermidis (MSSE) 35984 (MIC, 0.5 μg/mL). In addition to Staphylococcus, WYBQ-4 was also found to be active against Streptococcus pyogenes (MIC, 1 μg/mL), but the antibacterial activity against Enterococcus faecalis, Enterococcus faecium, and Gram-negative bacteria was weak compared to that against Staphylococcus.

### Remarkable antibacterial activity against clinical strains of S. aureus.

**FIG 2:**
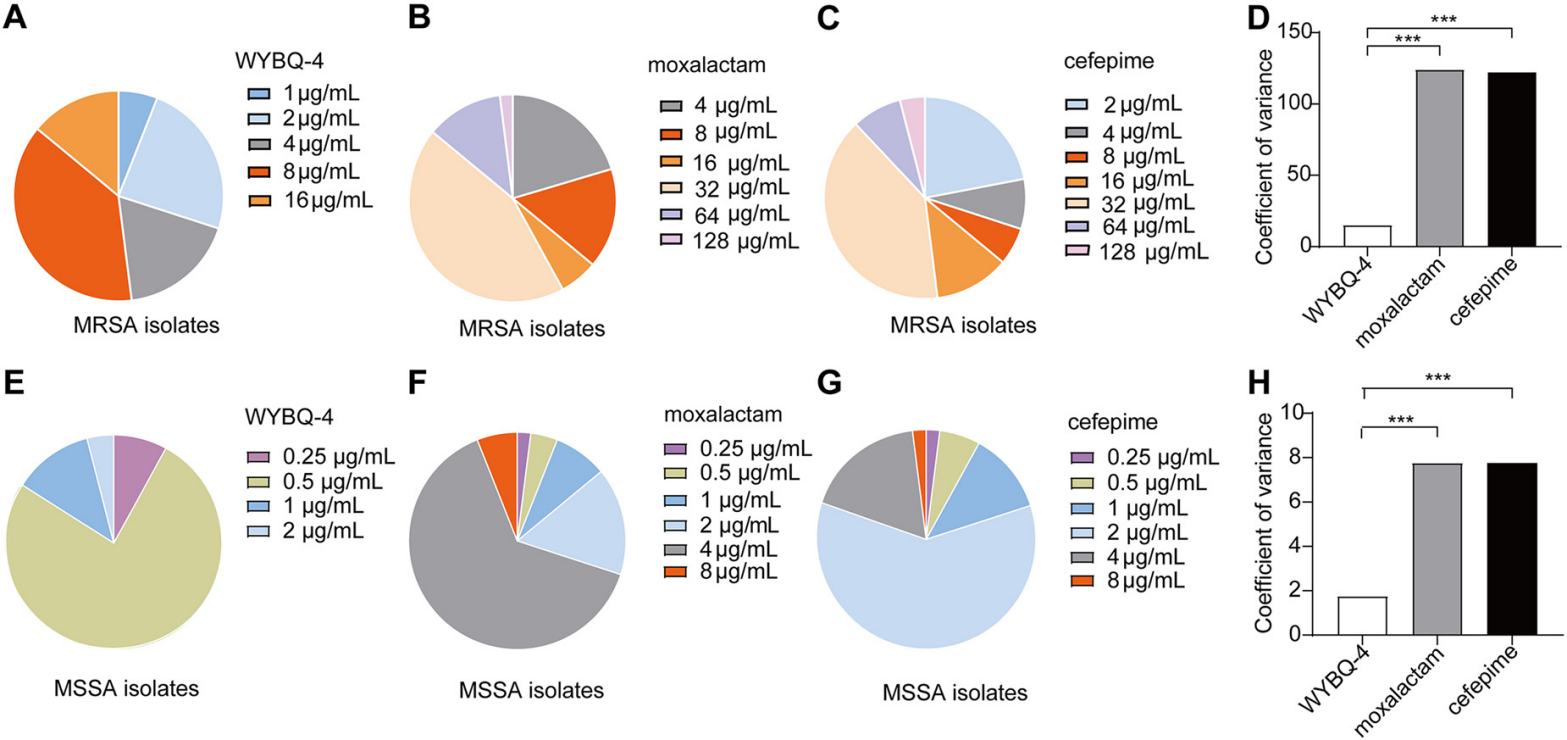
Antibacterial effects of WYBQ-4 on clinically isolated S. aureus strains. (A to D) The antimicrobial effect of (A) WYBQ-4, (B) moxalactam, or (C) cefepime on clinically isolated MRSA was determined by measuring the MIC and (D) the coefficient of variance of clinically isolated MRSA. (E to H) The antimicrobial effect of (E) WYBQ-4, (F) moxalactam, or (G) cefepime on clinically isolated MSSA was determined by measuring the MIC and (H) the coefficient of variance of clinically isolated MSSA.

Subsequently, we measured the MIC of WYBQ-4 and the antibiotics with definite clinical effects (moxalactam and cefepime) against clinically isolated MRSA and MSSA, respectively (10, 11). Among them, WYBQ-4 displayed a better bacteriostatic activity than the control drugs *in vitro* (Fig. 2A to C and E to G) and a smaller antibacterial coefficient of variance (Fig. 2D and H).

### Rapid eradication of MRSA USA300 and MSSA 29213 as determined by time-kill analysis.

The time-dependent killing effect of WYBQ-4 was evaluated against MSSA 29213 and MRSA USA300. Untreated S. aureus reached 10^9^ CFU/mL after culturing for 9 h from an initial period of 10^6^ CFU/mL and reached the stationary phase. According to the curves of the WYBQ-4-treated groups, obvious inhibition of bacterial growth was observed. When the concentration of WYBQ-4 reached 4× MIC, it showed complete sterilization on MSSA 29213 at 9 h (Fig. 1C), whereas MRSA USA300 treated with 8× MIC of WYBQ-4 showed complete sterilization at 9 h (Fig. 1D). Taking these data together, WYBQ-4 showed swift and radical bactericidal effects against MRSA ATCC 29213 and MRSA USA300 in a concentration-dependent manner.

### WYBQ-4 has a longer postantibacterial effect (PAE).

The results of the PAE of WYBQ-4 and moxalactam against MSSA ATCC 29213 are shown in Fig. 1E. The PAEs of WYBQ-4 to MSSA ATCC 29213 were 1.5 and 3.5 h, respectively, at 2× MIC and 8× MIC. However, the PAEs of moxalactam at 2× MIC and 8× MIC were 1 and 2.5 h, respectively. WYBQ-4 has a longer PAE than moxalactam.

### WYBQ-4 exhibits excellent nontoxicity.

**FIG 3:**
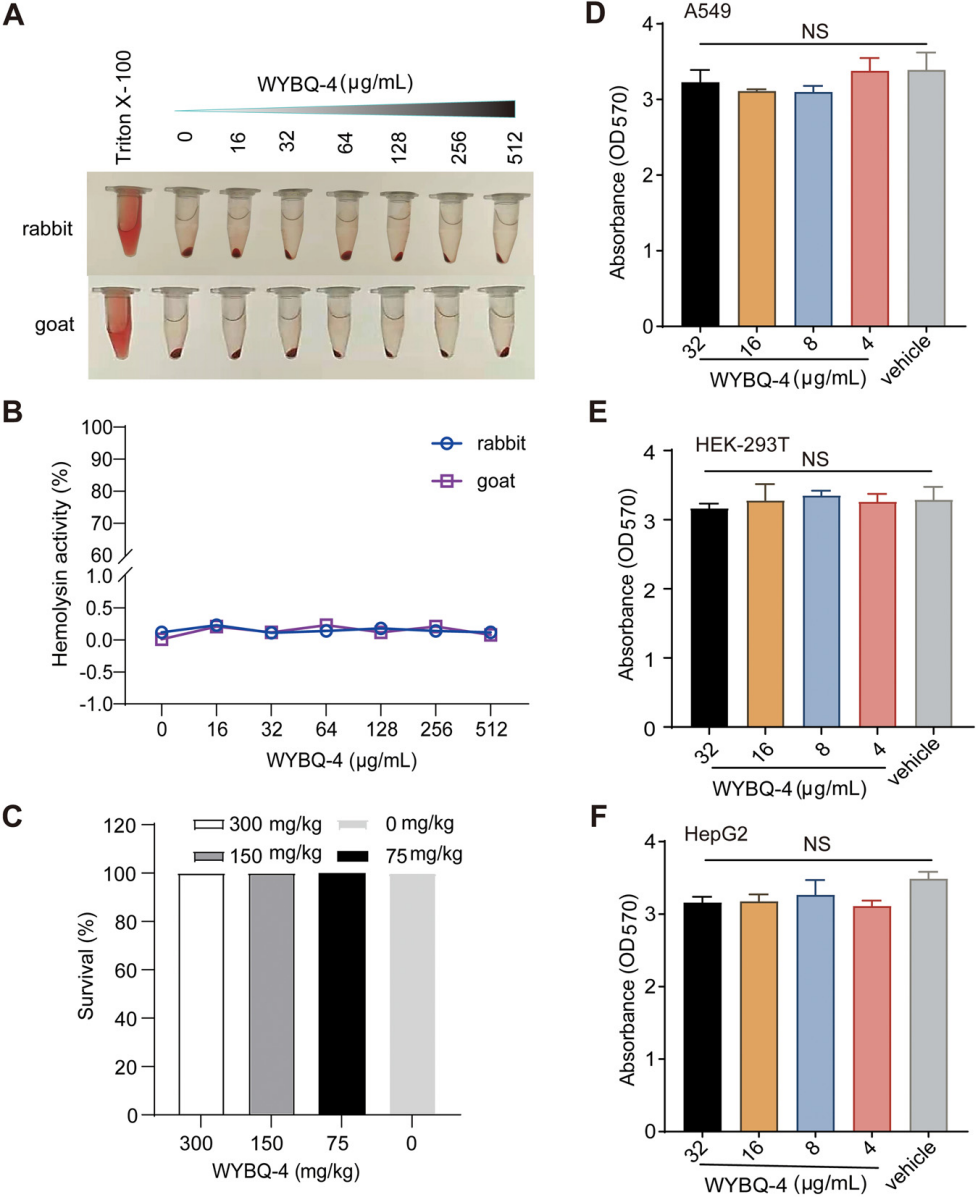
WYBQ-4 exhibits excellent safety. (A) The hemolytic activity assay revealed that different concentrations of WYBQ-4 did not destroy erythrocytes in rabbits and goats, and (B) the absorbance of the supernatant was measured at 543 nm. (C) The acute toxicity test showed that intraperitoneal injection of WYBQ-4 (300 mg/kg) did not affect the survival of the mice. (D to F) WYBQ-4 (0-128 μg/mL) did not affect the viability of A549, HEK-293T and HepG2 cells.

To assess the toxicity of WYBQ-4, we performed three independent experiments. First, hemolytic activity was measured using rabbit and goat red blood cells. WYBQ-4 did not show hemolytic activity in the concentration range of 0 to 512 μg/mL (Fig. 3A and B). Acute toxicity refers to the toxic effects caused by the body after a single exposure (or multiple exposures within 24 h) to foreign compounds, even causing death, and is one of the indicators for evaluating the safety of drugs. Subsequently, an acute toxicity assay of WYBQ was performed and showed no discomfort, delay, or inanimate behavior after intraperitoneal injection of 300, 150, or 75 mg WYBQ-4/kg body weight (Fig. 3C). The cytotoxicity of WYBQ-4 against A549, HEK-293T, and HepG2 cells was further assessed by methylthiazolyldiphenyl-tetrazolium bromide (MTT) assay, and the results showed that the cells were still viable at concentrations of 8 times the MIC against MRSA USA300 (Fig. 3D to F). In conclusion, these results indicate that WYBQ-4 was nontoxic at a concentration considerably higher than the MIC and is a promising antimicrobial drug for further development.

### Effect of WYBQ-4 on bacterial cell morphology.

**FIG 4:**
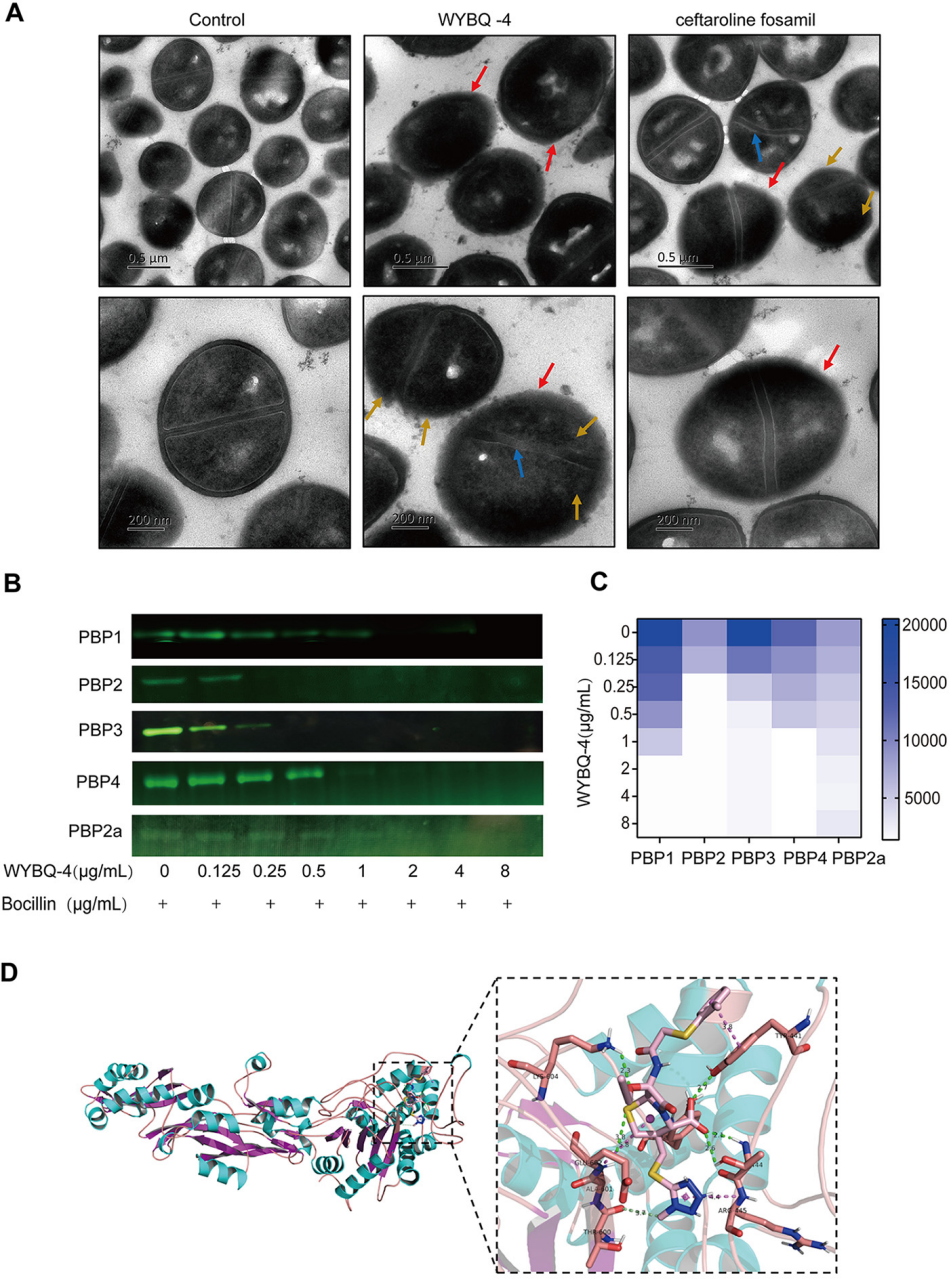
WYBQ-4 binds to PBP_S_ and thus affects the cell wall of MRSA. (A) Effect of WYBQ-4 on the cell morphology and ultrastructure of MRSA USA300. The main features are identified as follows: thin cell walls (red arrows), marked asymmetry of bacterial division (yellow arrows), and distortion of septa (blue arrows). (B and C) Direct binding of WYBQ to PBPs (PBP1 to 4, 2a) was confirmed by a bocillin assay (B) and grayscale analysis (C). (D) Molecular docking simulates the key amino acid binding site between WYBQ-4 and PBP2a.

Transmission electron microscope (TEM) assays were performed to further investigate the effect of WYBQ-4 on the morphology of S. aureus. As shown in Fig. 4A, untreated MRSA had normal morphology with distinct septa. After exposure to 1/2× MIC WYBQ-4 for 3 h, we observed thin cell walls (red arrows), marked asymmetry of bacterial division (yellow arrows), and distortion of septa (blue arrows). Then, the quantification of division defects in different groups was also analyzed and is presented in Table 1. When we scanned thin sections of 64 cells from USA300 strains, about 23.4% of the bacteria showed cell wall thinning, which could be related to bacterial proliferation or physical damage during sample processing. In contrast, 71.9% and 63.4% of cell wall dissipation occurred in the ceftaroline and WYBQ-4 groups, respectively, which were significantly higher than in the untreated group (wild type [WT]) (*P < *0.01). Similar results were also observed in bacterial asymmetric division and distortion of septa. In conclusion, these results indicated that WYBQ-4 causes cell wall thinning, septum distortion, and division asymmetry in S. aureus.

**TABLE 1:** Analysis of cell morphological changes in different treatment groups^*a*^

Group	Thin cell walls	Asymmetry of bacterial division	Distortion of septa	Total number of cells	Percentage of defective cells (thin cell walls) (%)	Percentage of defective cells (asymmetry of bacterial division) (%)	Percentage of defective cells (distortion of septa) (%)
WT	15	6	5	64	23.4	9.38	10.9
Ceftaroline fosamil	64	41	31	89	71.9 C	46.1 C	34.8 B
WYBQ-4	59	38	27	93	63.4 c	40.9 b	29.0 a

a Uppercase B represents a significant difference between the ceftaroline fosamil and WT groups (*P* < 0.01). Uppercase C represents an extremely significant difference between the ceftaroline fosamil and WT groups (*P* < 0.001). Lowercase a represents a significant difference between the WYBQ-4 and WT groups (*P* < 0.05). Lowercase b represents a significant difference between the WYBQ-4 and WT groups (*P* < 0.01). Lowercase c represents a significant difference between the WYBQ-4 and WT groups (*P* < 0.001).

### WYBQ can specifically bind to PBPs of MRSA.

To explore the antibacterial mechanism, the binding affinities of WYBQ-4 for PBP1, PBP2, PBP3, PBP4, and PBP2a of S. aureus USA300 were determined using a competition fluorescence gel assay, and bocillin was used as the fluorescent ligand. As shown in Fig. 4B and C, the fluorescence intensity of PBPs gradually decreased with increasing drug concentration, which indicated that WYBQ-4 had a certain affinity for PBPs. Among them, WYBQ-4 has a certain affinity for PBP2, as the fluorescence disappeared completely when 0.25 μg/mL WYBQ-4 was added. In addition, we observed that 2 μg/mL WYBQ-4 completely competed for the binding of bocillin to PBP2a, which mediates the resistance of MRSA to most β-lactam drugs. The results imply that WYBQ-4 can achieve antimicrobial effects by binding to PBPs of MRSA and subsequently affect the transpeptidase activity, ultimately interfering with cell wall synthesis.

### Molecular docking reveals a potential interaction between WYBQ-4 and PBP2a.

Since there is a direct interaction between WYBQ-4 and PBP2a, we simulated the key site and binding mode of WYBQ-4 binding to PBP2a by molecular docking. As illustrated in Fig. 4D, WYBQ-4 can bind stably to the pocket of PBP2a protein, where TYR441, THR444, TYR519, ALA601, GLU602, and LYS604 are the key amino acid sites for PBP2a to bind to WYBQ-4. The hydrophobic interaction force, hydrogen bonding, contributes to the binding of both, and the total binding free energy was calculated to be −8.1 kcal/mol, confirming a strong interaction of WYBQ-4 with PBP2a.

### Kinetic parameters of the reaction of WYBQ-4 with PBP2a.

The kinetics of WYBQ-4’s interactions with PBP2a have been evaluated. The nonspecific secondary rate constant, *k_0_*, was firstly derived from the linear phase of absorbance variation with time, and then the apparent primary rate constant of the acylation reaction of nitrofuran at 100 μM was determined by absorbance variation with time. This procedure was calculated using the WolframAlpha website (https://www.wolframalpha.com/). The calculated secondary rate constant, *k_0_*, for the nonspecific hydrolysis of nitrocefin is 2.3 ± 0.14 M^−1^s^−1^; the primary reaction rate constant, kn*, for 100 μM nitrocefin is approximately 0.05 s^−1^.

Subsequently, nitrocefin (100 μM) was used as a reporter molecule to determine the apparent first-order rate constants for WYBQ-4 acylation at different concentrations (0 to 500 μM) in competition assays. Since the modified WYBQ-4 is based on the structure of cephamycin C, it should have an effect similar to that of other cephalosporin antibiotics, for which the acylation rate constant (*k2*) is large but deacylation (*k3*) is very small (12). The results are shown in Table 2. The second-order rate constant, *k_2_*/*K_s_*, for PBP2a acylation by WYBQ-4 is derived from the linear regression of the observed rate of acylation (ki) with *k_2_*/*K_s_* of 174 ± 8.45 M^−1^s^−1^. In addition, the second-order rate constant for the PBP2a acylation by ceftaroline has been reported to be 4,500 M^−1^s^−1^. It is possible that ceftaroline’s k2/*K_s_* ratio was greater because conformational changes enabled it to open the active site of PBP2a, resulting in a more favorable acylation reaction. Subsequently, PBP2a (15 μM) and WYBQ-4 (0 to 90 μM) were included in the 60-μL reaction system for 45 min, and the free protein was detected by adding BOCILLIN FL when the reaction reached equilibrium, and the *K_s_* was found to be 28 ± 1.24 μM.

**TABLE 2:** Kinetics parameters for interactions of *β*-lactam antibiotics with PBP2

β-Lactam	*k*_2_ (s^–1^ × 10^3^)	*ks* (μM)	*k*_2_/*ks* (M^–1^s^–1^)	*k_3_* (s^–1^ × 10^6^)	Reference
WYBQ-4	4.872 ± 0.14	28 ± 1.24	174 ± 8.45	11±1.32	
Ceftaroline		20 ± 4	4,500 ± 640	—	47
Nitrocefin	3.7 ± 0.3	192 ± 24	19.0 ± 3.0	7.2 ± 0.1	48
Cefepime	1.5 ± 0.1	1,618 ± 145	0.9 ± 0.1	5.9 ± 0.5	48
Ceftazidime	1.0 ± 0.1	671 ± 116	1.5 ± 0.3	3.2 ± 0.2	48
Ampicillin	3.4 ± 0.1	668 ± 124	5.0 ± 1.0	3.2 ± 0.1	48

In addition, we also observed that WYBQ-4 exhibited a lower rate of deacylation of PBP2a, similar to other β-lactam antibiotics, with a *k3* of 11 ± 1.32 s^−1^ × 10^6^. In conclusion, the high acylation rate and low deacylation rate of PBP2a exhibited by WYBQ-4, coupled with its better antibacterial ability, has the potential for further development.

### Protective effect of WYBQ-4 on S. aureus-induced pneumonia in mice.

**FIG 5:**
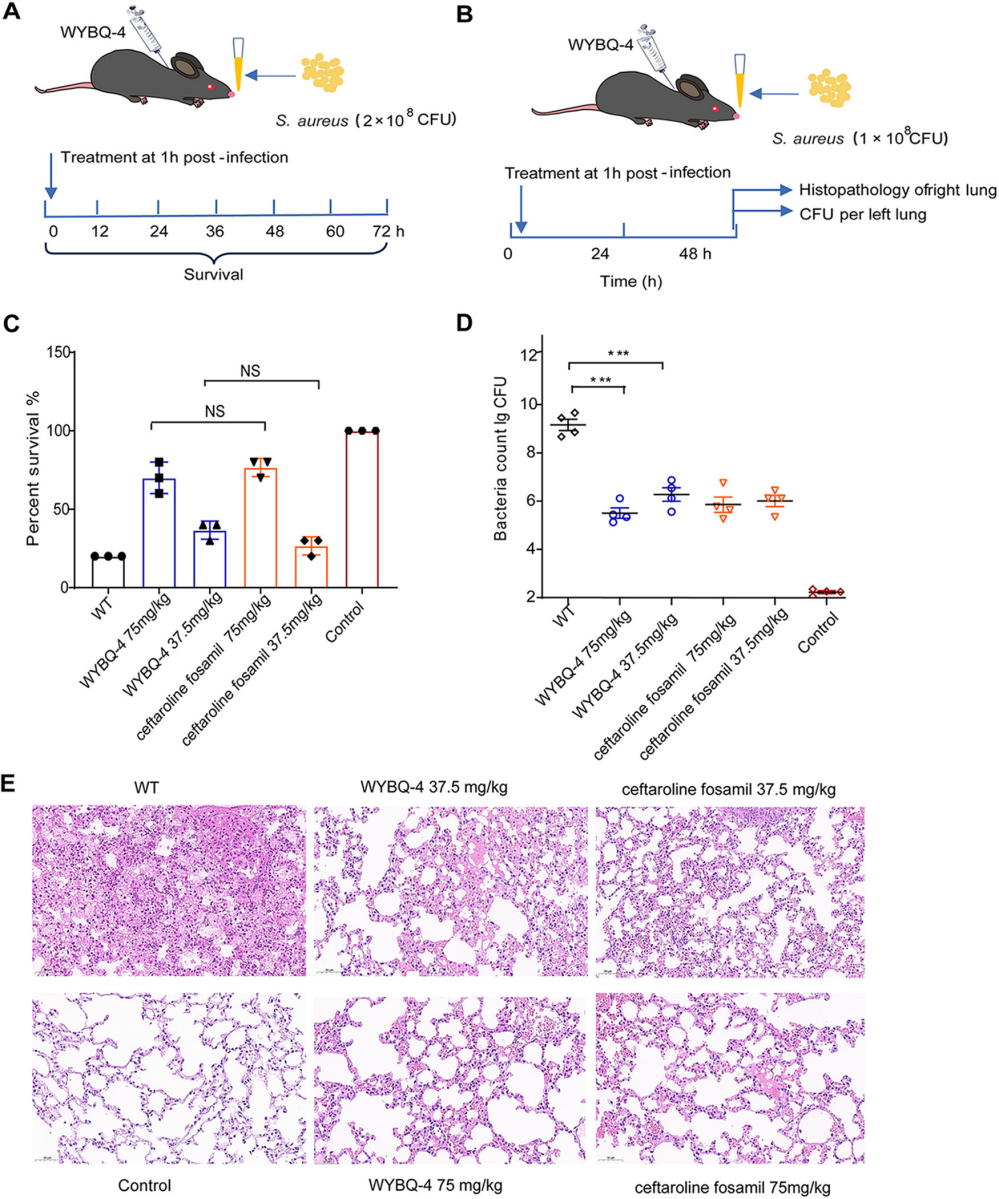
WYBQ-4 protects mice from S. aureus-induced pneumonia. (A and B) Scheme of (A) survival and (B) lung tissue and bacterial load in a mouse pneumonia infection model. (C) Effect of WYBQ-4 and ceftaroline fosamil treatment on the survival of mice (*n* = 10) infected with a lethal dose of S. aureus within 72 h. Log-rank test; NS, no significant difference (*P > *0.05). (D) Effect of WYBQ-4 treatment on the bacterial load in the lungs of mice (*n* = 4). ***, *P* < 0.001 versus the WT group; Mann-Whitney test, two-tailed. (E) Histopathology of the lungs (H&E stained) of mice treated with or without WYBQ-4. Scale bar = 50 μm.

To evaluate the therapeutic activity of WYBQ-4 in the lung model, mice were challenged with USA300 (2 × 10^8^ CFU) and then treated with WYBQ-4 (75 and 37.5 mg/kg) (Fig. 5A). Untreated mice began to die 12 h after inoculation with USA300, and the survival rate within 72 h was 20%. After treatment with 75 and 37.5 mg/kg WYBQ-4, the survival rates of the mice were 70.00% and 36.67%, respectively (Fig. 5C). For the positive-control group, the survival rates of mice treated with 75 and 37.5 mg/kg of ceftaroline fosamil were 76.67% and 26.67%, respectively. These results showed that both WYBQ-4 and ceftaroline fosamil significantly improved the survival rate of mice with S. aureus pneumonia with no significant difference (*P > *0.05).

The lung tissue bacterial load of WYBQ-4-treated mice was also further assessed as shown in Fig. 5B. The MRSA USA300 count in the lung was 9.45 ± 0.21 log_10_ CFU/g in the infected mice without treatment. After treatment with 75 mg/kg WYBQ-4, the bacterial counts in the lung were reduced significantly to 5.44 ± 0.15 log_10_ CFU/g compared with untreated mice (Fig. 5D). There was no significant difference in the therapeutic effect between WYBQ-4 and ceftaroline fosamil in the range of phase doses (*P > *0.05). Thus, 75 mg/kg WYBQ-4 significantly reduced the number of surviving MRSA USA300 organisms in the lungs of mice.

To investigate whether WYBQ-4 can ameliorate the lung damage caused by nonlethal exposure to USA300, the degree of damage to the lungs for different doses of WYBQ-4 was observed. Untreated infected mice showed some degree of acute injury characterized by interstitial hyperemia and edema with marked focal hemorrhage. After treatment with WYBQ-4 or ceftaroline fosamil, inflammatory signs of lung tissue were significantly reduced, with little infiltration of inflammatory cells. Moreover, WYBQ-4 (75 mg/kg) showed better efficacy than ceftaroline fosamil (75 mg/kg) in the treatment of infected lesions (Fig. 5E).

Taken together, these data indicate that WYBQ-4 has a significant therapeutic effect against S. aureus-induced pneumonia.

### Protective effect of WYBQ-4 on S. aureus-induced intramuscular infection in mice.

**FIG 6:**
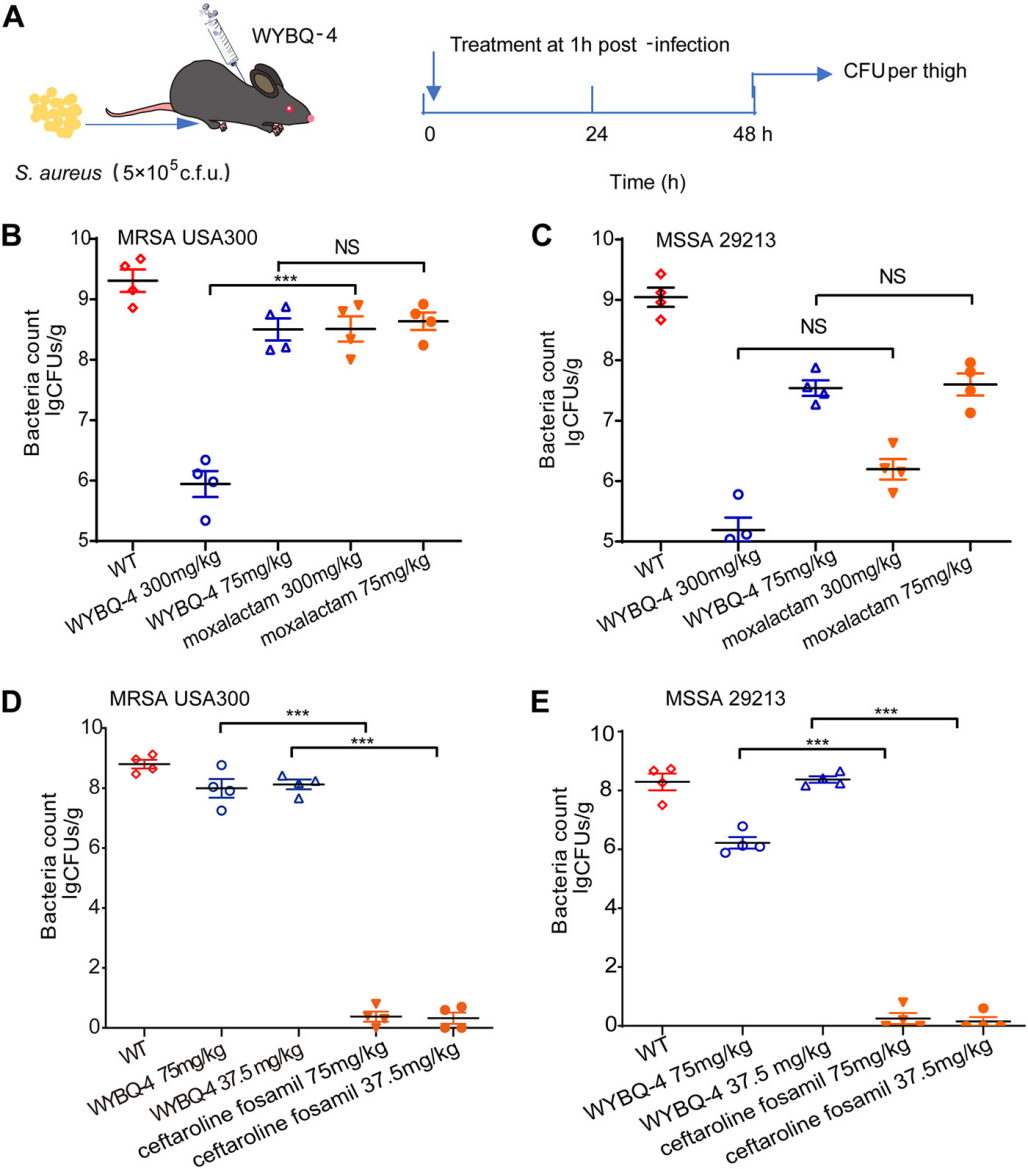
WYBQ-4 protects mice from S. aureus*-*induced intramuscular infection. (A) Experimental protocol for the mouse intramuscular infection model. (B and C) Effects of (B) WYBQ-4 (300 and 75 mg/kg) and moxalactam (300 and 75 mg/kg) on the bacterial load of MRSA USA300 and (C) MSSA 229213 on the thighs of mice. (D and E) Effect of (D) WYBQ-4 (75 and 37.5 mg/kg) and ceftaroline fosamil (75 and 37.5 mg/kg) on the bacterial load of MRSA USA300 and (E) MSSA 229213 on the thighs of mice. NS, no significant difference (*P > *0.05), ***, *P* < 0.001 versus the WT group. Mann-Whitney test, two-tailed.

The therapeutic effects of WYBQ-4 on intramuscular infections induced by MRSA USA300 and MSSA 29213 in mice were evaluated. Mice were given S. aureus (5 × 10^5^ CFU) via thigh injection, followed by subcutaneous administration of WYBQ-4 (300, 150, 75, and 37.5 mg/kg) treatment (Fig. 6A). As shown in Fig. 6B to E, the bacterial loads in the thighs of MRSA USA300- and MSSA 29213-infected mice were 9.33 log_10_ CFU/g and 9.12 log_10_ CFU/g, respectively. After treatment with WYBQ-4 (300 mg/kg), the bacterial load decreased to 5.97 log_10_ CFU/g and 5.21 log_10_ CFU/g, respectively. WYBQ-4 (300 mg/kg) exhibited more prominent activity in removing bacteria from thigh tissue than moxalactam (300 mg/kg) in both the MRSA and MSSA infection groups (Fig. 6B and C). However, the treatment effect of WYBQ-4 was ineffective compared to that of ceftaroline fosamil in this infection model.

### Protective effect of WYBQ-4 on S. aureus-induced systemic infection in mice.

**FIG 7:**
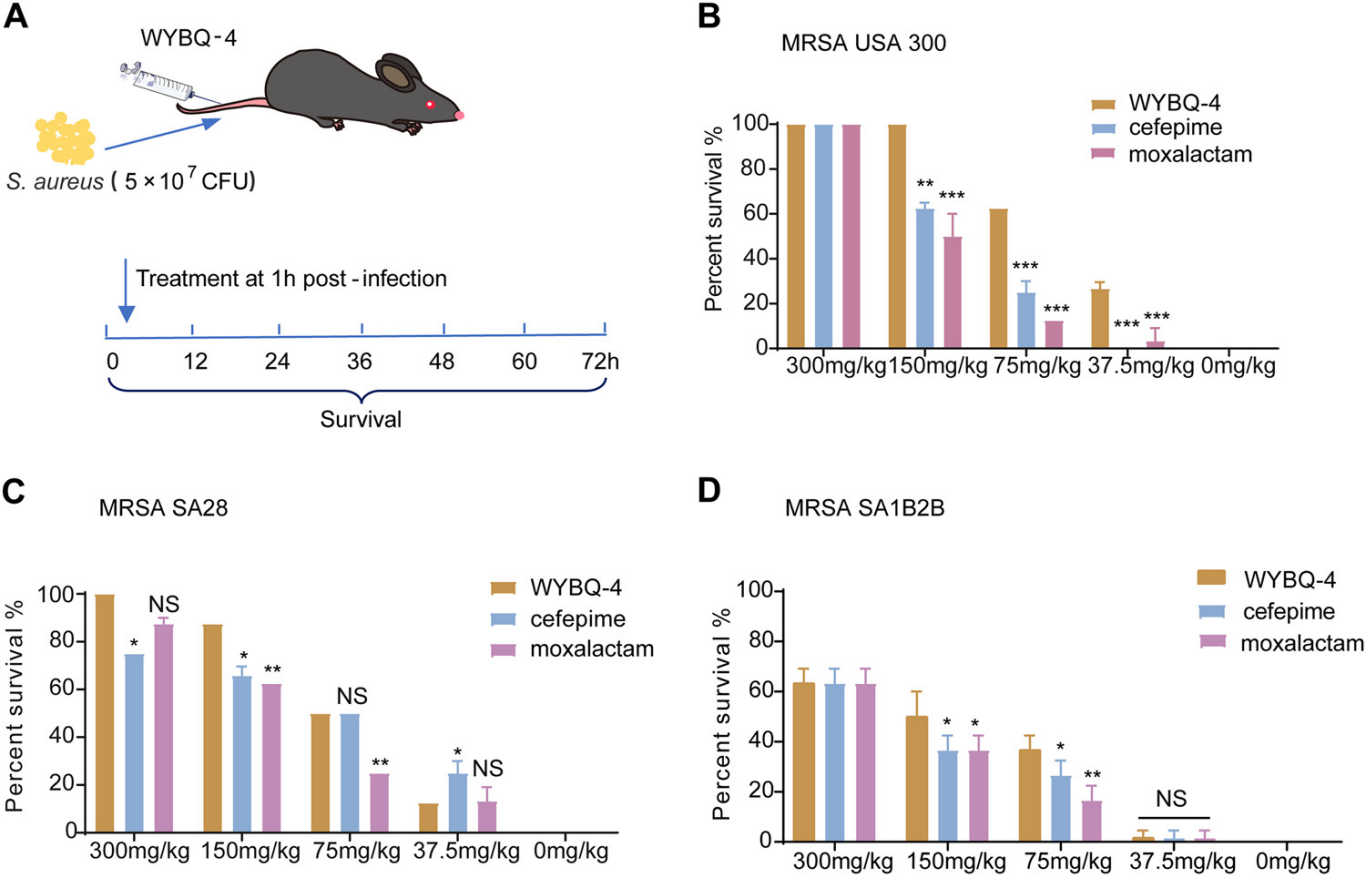
Protective effect of WYBQ-4 on systemic infection induced by MRSA USA300 and clinically isolated MRSA strains in mice. (A) Experimental protocol for a mouse systemic infection model. (B to D) Protective effects of WYBQ-4, cefepime, and moxalactam against systemic infection induced by (B) MRSA USA300 and clinical isolates of MRSA strains, (C) SA28, and (D) SA1B2B in mice.

To assess the therapeutic effect of WYBQ-4 on a mouse systemic infection model, mice were challenged with USA300 (5 × 10^7^ CFU) followed by treatment with WYBQ-4 (300, 150, 75, and 37.5 mg/kg) (Fig. 7A). The survival rate of mice treated with 150 mg/kg WYBQ-4 was 100%. However, the survival rate of mice treated with cefepime or moxalactam at the same dosage was 40% and 50%, respectively (Fig. 7B). For the clinical isolate SA28 and the SA1B2B systemic infection mouse model, WYBQ-4 also had a protective effect at different doses (Fig. 7C and D). Taken together, the efficacy of WYBQ-4 in treating systemic infections caused by S. aureus in mice is higher than that of cefepime and moxalactam.

## DISCUSSION

The emergence, prevalence, and widespread distribution of multidrug-resistant pathogens have caused enormous economic losses and health problems worldwide. This is dominated by S. aureus, which has developed multiple strategies to combat antibiotics. For example, MRSA resistance to β-lactam antibiotics is attributed to the involvement of the *mecA*-encoded PBP2a in cell wall synthesis, which has a low affinity for β-lactam antibiotics (13). In addition, bacteria evade antibiotics in a dormant state of nongrowth (14) or by forming biofilms (15), overexpressing efflux pumps, and in many other ways. These factors lead to a high morbidity and mortality rate of MRSA infections, which are extremely difficult to treat and have emerged as one of the current dilemmas in the treatment of surgical infections. Therefore, searching for new and potent antibacterial drugs for the treatment of MRSA infections has become a current priority.

WYBQ-4 (16) is a semisynthetic antibiotic of cephamycin C as a precursor to synthesize methoxy cephalosporins. Cefoxitin, cefotetan, cefmetazole, and temocillin also belong to this class of antibiotics (17) and have been widely used in the clinical treatment of a variety of bacterial infectious diseases (18). They have a trans-methoxy at the 7α-position on the β-lactam ring, which results in strong stability against β-lactamase. In contrast, WYBQ-4 was obtained from the third generation of cephalosporin antibiotics based on the modification and optimization of sites I and III. The introduction of a heterocycle at the 1st position can improve the steric hindrance of the β-lactam ring and the stability of the chemical bond, thereby increasing the stability of the β-lactamase, increasing the antibacterial spectrum, and improving its antibacterial activity. Position III is the 3-position substituent of the parent nucleus, and its modification not only affects the antibacterial activity and antibacterial spectrum of the drug but also affects the pharmacodynamics and pharmacokinetic properties of the drug. Among a series of optimized compounds, WYBQ-4 showed a strong antibacterial effect against MRSA, and further exploration of its antibacterial effect and mechanism of action is of great significance for the investigation and exploration of novel agents.

In our study, the antimicrobial spectrum of WYBQ-4 was evaluated by measuring the MIC, which is the most used criterion for assessing the susceptibility of bacteria to drugs and is the basis for clinicians to select appropriate antimicrobial drugs (19). The results showed that WYBQ-4 had remarkable antibacterial effects against S. aureus and against clinical isolates of MRSA and MSSA (Fig. 2). A time-kill curve assay also confirmed the rapid bactericidal ability of WYBQ-4 against MSSA and MRSA.

Additionally, PAE is an essential pharmacodynamic parameter when choosing an antibiotic dosing regimen in clinical practice (20, 21). The results showed that WYBQ-4 has a longer PAE against S. aureus than moxalactam. Longer PAEs may lead to longer dosing intervals, thus potentially reducing treatment costs and inhibiting the emergence of drug resistance (22). Furthermore, the cytotoxicity, hemolysis, and acute toxicity assays proved that WYBQ-4 has excellent safety and can be further developed as a new anti-S. aureus infection agent.

S. aureus can express four natural PBPs (PBP1, PBP2, PBP3, and PBP4) (23, 24). Furthermore, MRSA expresses a fifth PBP (PBP2a), which, unlike natural S. aureus PBPs, is a poor target for β-lactam antibiotics (25, 26). As a family of proteins, PBPs are involved in the final stages of bacterial cell wall formation, especially the division of dividing cells (27–29). We first analyzed the changes in the morphology of S. aureus after WYBQ-4 treatment. Untreated S. aureus looked normal, with peptidoglycan and cytoplasmic membrane clearly visible. However, when MRSA was exposed to WYBQ-4, it resulted in the disappearance of cell walls and marked inhibition of bacterial division with uneven division.

BOCILLIN FL is able to bind to PBPs specifically and has been widely used to identify the affinity between small-molecule drugs and PBPs (30). Subsequently, the affinity between PBP1 to 4 and WYBQ-4 was further evaluated using bocillin, and the results showed that WYBQ-4 had an affinity for PBP1 to 4 in a dose-dependent manner. Among them, the affinity with PBP2 is stronger than that with the other three. Moreover, WYBQ-4 had a strong affinity for PBP2a, with a 50% inhibitory concentration (IC_50_) of 2 μg/mL, which is significantly better than that of ceftriaxone (IC_50_, 677 ± 53 μg/mL) and oxacillin (IC_50_, 408 ± 6 μg/mL), as reported (31). Furthermore, the IC_50_ of WYBQ was comparable to that of ceftriaxone (1.6 μg/mL) for PBP2a (31). A stronger affinity of antibiotics for PBP2a means a better anti-MRSA effect (29). Subsequently, molecular docking showed a total binding free energy of −8.1 kcal/mol for WYBQ-4 with PBP2a, demonstrating a prominent interaction. In summary, the above-described results demonstrated that the remarkable anti-S. aureus, especially the anti-MRSA, effect of WYBQ-4 was most appropriate due to its binding activity to PBPs (PBP1 to 4 and PBP2a), further inhibiting the transpeptidation and synthesis of the bacterial cell wall.

S. aureus is a medically important pathogenic bacterium. It can cause extensive purulent damage involving multiple organs in humans and animals, including skin infections, pneumonia, endocarditis, toxic shock syndrome, systemic infections, and septic arthritis, and is a major source of hospital outbreaks and community-acquired infections (32–34). Ceftaroline fosamil, cefepime, and moxalactam are commonly used drugs for the treatment of complicated skin and soft tissue infections, systemic infections, and bacterial pneumonia in clinical practice (35). To this end, we evaluated the therapeutic efficacy of WYBQ-4 and commonly used clinical antibiotics against MRSA-induced pneumonia, systemic infections, and intramuscular infection models in mice. In our experiments, it was observed that WYBQ-4 and ceftaroline fosamil have similar effects on the mouse pneumonia infection model at the same dose. WYBQ-4 treatment improved the survival rate of mice, reduced the bacterial load of lung tissue, and alleviated lung damage. In the local infection model, WYBQ-4 was slightly less effective than ceftaroline fosamil in treatment. WYBQ-4 exhibited remarkable therapeutic effects on MRSA USA300 and clinically isolated MRSA-induced systemic infections compared to cefepime and moxalactam and significantly improved the survival rate of mice. In summary, these results suggest that WYBQ-4 is suitable for the treatment of pneumonia and systemic infections caused by MRSA.

The remarkable antimicrobial effect of WYBQ-4 *in vivo* and *in vitro* suggested that it could be a new candidate compound for the control of MRSA transmission and the treatment of MRSA infections.

## MATERIALS AND METHODS

### Strains, chemicals, plasmids, and growth conditions.

Detailed bacterial strain information is provided in Table S1. The clinical strains of MRSA and MSSA isolated in Beijing, Sichuan, and Shanghai in the last 3 years totaled more than 300 strains. Clinical isolates were stored at −80°C in 20% glycerol-rich brain heart infusion (BHI) and passaged twice on Trypticase soy agar prior to the experiment. Before the experiment, 16s RNA identification was performed, and then PCR was used to distinguish MRSA and MSSA by identifying the *mecA* gene. Furthermore, the MIC of cefoxitin against clinical isolates was tested again. The drug sensitivity results were judged according to the criteria established by the American Society for Clinical Laboratory Standardization. MRSA was determined by a cefoxitin MIC of ≥8 μg/mL (36). Finally, 100 strains each of MRSA and MSSA were randomly selected from the identified strains for subsequent experiments. All chemical reagents and antibiotics were purchased from Sigma (St. Louis, MO). S. aureus was grown at 30°C in BHI broth (Oxoid) with constant shaking or on BHI agar plates unless otherwise noted. Escherichia coli was grown overnight in lysogeny broth (LB; Oxoid) or on LB plates at 37°C. When required for selective growth of S. aureus, 10 μg/mL chloramphenicol (Cm) and erythromycin (Em) were added to the culture medium. For E. coli, 50 μg/mL kanamycin (Kan) was used for selection. WYBQ-4 is a new type of cephalosporin antibiotic with a molecular formula of C_22_H_22_N_6_O_5_S_3_ (16). The data of ^1H^ NMR and high-resolution mass spectrometry (HRMS; ESI) are presented in the Fig. S1 and S2.

### Antimicrobial activity.

The antibacterial spectrum and susceptibility of WYBQ-4 against MRSA USA300, MSSA 29213, and clinically isolated S. aureus were determined using a broth microdilution method following the guidelines outlined by the Clinical and Laboratory Standards Institute (CLSI) (37, 38). Briefly, 96-well plates containing different concentrations of WYBQ-4 and 10^5^ CFU of bacteria in 100 μL of cation-adjusted Mueller-Hinton broth (CAMHB) were incubated at 37°C for 16 h prior to determining the MIC. The absorbance was measured at 600 nm, and experiments were repeated in three biological replicates.

### Time-kill curve.

The bactericidal activities of WYBQ-4 against MSSA 29213 and MRSA USA300 were determined *in vitro*. Overnight cultures were diluted to 10^6^ CFU (CFU)/mL in 10 mL of fresh Mueller-Hinton broth (MHB) medium. Different amounts of antibiotics were added so that the final concentrations were equivalent to 0.5× MIC, 1× MIC, 2× MIC, 4× MIC, 8× MIC, and 16× MIC of WYBQ-4. After incubation at 37°C with shaking for 0 to 24 h, 100-μL samples from each time point were subcultured on MHB agar plates and incubated at 37°C for 24 h. MSSA 29213 and MRSA USA300 without WYBQ-4 were used as control groups. Colony numbers were counted to calculate the CFU, and the data were expressed as log_10_ CFU. Three biological replicates were performed for the experiment.

### Postantibiotic effect.

The postantibiotic effect (PAE) of WYBQ-4 against MSSA 29213 was determined in MHB agar using the viable plate count method (39). Briefly, 5 mL of S. aureus (10^6^ CFU/mL) was supplemented with final concentrations of 2× MIC and 8× MIC of WYBQ-4. After incubation at 37°C for 1 h, WYBQ-4 was removed by centrifugation and washing with phosphate-buffered saline (PBS). Subsequently, fresh MHB medium was added to resuspend the bacteria, and the mixture was incubated at 37°C with shaking. The number of colonies was counted by taking 10 μL of bacteria at 1-h intervals and applying them to BHI agar plates after appropriate dilution. PAE duration was calculated as follows: time (h) for bacteria to return to log-phase growth after drug treatment – time (h) for the control group to return to log-phase growth (40).

### Safety assessment.

**Cytotoxicity assay.** The cytotoxicity of WYBQ-4 against A549, HEK-293T, or HepG2 cells was evaluated with a cell counting kit-8 kit (CCK8; US Everbright, Suzhou, China) according to the manufacturer’s instructions (41). In brief, 5 × 10^4^ cells were seeded in 96-well plates containing prewarmed medium (100 μL) and incubated for 24 h at 37°C in 5% CO_2_. Then, various concentrations of WYBQ-4 (0 to 512 μg/mL) were added and further incubated for 24 h. Afterward, the cells were incubated with 100 μL of medium with 10 μL of CCK8 solution (Sigma-Aldrich) for 4 h. Finally, a solution of the purple precipitate dissolved in dimethyl sulfoxide (DMSO) was added to a 96-well plate, and the absorbance was measured at 450 nm.

**Hemolysin activity.** Rabbit blood cells (RBCs) and goat blood cells (GBCs) were washed three times with sterile PBS, centrifuged (4,000 × *g*, 5 min) to collect the blood cells, and then resuspended in PBS to obtain an 8% blood cell suspension. The blood cells were mixed with various concentrations of WYBQ-4 ranging from 0 to 512 μg/mL (0.2% vol/vol). Triton X-100 with the same treatment was used as a control. After incubation at 37°C for 1 h, the samples were centrifuged (4,000 × *g*, 5 min), and the absorbance of the supernatant was measured using a microplate reader at a wavelength of 570 nm.

**Acute toxicity test in mice.** The acute toxicity test in mice was performed according to the requirements of the Chinese Guidelines for Acute Toxicity of Chemicals (H-GPT1-1) (42). In brief, each group of 6 female mice (6 to 8 weeks old, weights of 18 to 20 g, BALB/c) was intraperitoneally injected with a single dose of 300, 150, or 75 mg/kg body weight of WYBQ-4. The mice were injected with PBS as a control group. Then, the poisoning symptoms, abnormal behavior, and survival of the mice in each group were observed within 72 h.

### Transmission electron microscopy (TEM) for S. aureus visualization.

MRSA USA300 was cultured overnight, diluted (1:100) into 20 mL of Trypticase soy broth (TSB) medium, and incubated at 30°C with shaking until the optical density at 600 nm (OD_600_) reached 0.3. After addition of 4 μg/mL (1/2 of their respective MIC) WYBQ-4, the culture was shaken at 30°C for 3 h. Bacterial cells treated with 4 μg/mL ceftaroline fosamil (1/2 of their respective MIC) served as a control group. Untreated S. aureus served as a negative control. Bacteria were collected and washed in PBS and resuspended in 4% (vol/vol) glutaraldehyde fixation overnight. The fixed samples were further processed, and morphological changes in S. aureus were observed by TEM (JSM-7900F; JEOL, Japan).

### Preparation of bacterial membrane proteins.

Using the genome of S. aureus USA300 as a template, PCR was performed to amplify PBP1, PBP2, PBP3, PBP4, and PBP2a genes with primers designed according to Table 1. Then, the amplified fragments were treated with BamHI/XhoI and then inserted into the cloning site of the BamHI/XhoI-digested pET28a to yield pET28a-PBP1, pET28a-PBP2, pET28a-PBP3, pET28a-PBP4, and pET28a-PBP2a, respectively. The plasmid was transformed into E. coli BL21(DE3) and cultured in LB medium containing Kan resistance until the OD_600_ reached 0.6, and PBP expression was induced overnight at 16°C by adding 0.5 mM IPTG (isopropyl-β-d-thiogalactopyranoside). The bacteria were then lysed by sonication, the pellet was collected, and the proteins were purified using a HIS-Select nickel affinity gel system (Beyotime, Shanghai, China).

### PBP binding assays.

PBP binding assays were performed as previously described (30, 31). Selective labeling of PBP from preincubated samples (10 mg protein) with various concentrations of WYBQ-4 was performed for 15 min, and then 25 μM BOCILLIN FL (Invitrogen) was added, followed by an additional 10 min at 37°C. Proteins were separated by SDS-PAGE, and the PBPs were detected by a gel fluorescence imaging instrument (excitation, 488 nm; emission, 530 nm).

### Molecular docking.

The crystal structure of the PBP2a protein was obtained from the PDB database (https://www.rcsb.org/) with PDB ID 1VQQ and a resolution of 1.80 Å. Subsequently, the protein was preprocessed using Discovery Studio software to remove water molecules, hydrogenation, and charges, extract the proligands from the structure, and visualize the processed protein using PyMOL software. WYBQ-4 is docked with 1VQQ using the active site docking mode, which is centered at center-x_29.481, center-y_28.946, and center-z_86.704. Molecular docking was performed by applying AutoDock Vina software to predict the interaction and energy between WYBQ-4 and PBP2a.

### Determination of the kinetic parameters for interactions of WYBQ-4 with PBP2a.

Please refer to the supplemental material for the kinetic parameters of the interaction between WYBQ-4 and PBP2a and the determination of the rate of diacylation.

### Mouse lung infection model.

The animal experiments were performed using 6-week-old male C57BL/6J mice, and the mouse model of lung infection caused by MRSA USA300 was established as previously described (43, 44). For the survival rate, mice (10 per group) were infected with MRSA USA300 (2 × 10^8^ CFU per mouse). After 2 h of infection, mice were given WYBQ-4 via subcutaneous injection and reinjected at 12-h intervals, whereas mice were treated with ceftaroline fosamil at the same doses and served as positive controls. Mice injected with only MRSA USA300 were used as a negative control. Survival of mice was recorded within 72 h, and survival rates were calculated.

The mice were challenged with 1 × 10^8^ CFU of MRSA USA300 to assess bacterial load and pathological changes. The dose and manner of administration in mice were consistent with the survival experiments. In addition, the mice treated with ceftaroline fosamil at 75 and 37.5 mg/kg were used as control groups. At 48 h postinfection, mice were euthanized, and the left lungs were aseptically removed, weighed, and homogenized. Then, the lung tissues were homogenized and serially diluted for CFU counts. The right lungs were fixed with 10% formalin, embedded in paraffin, thin-sectioned, subjected to conventional hematoxylin and eosin (H&E) staining, and then observed using an optical microscope.

### Mouse intramuscular infection model.

The therapeutic effects of WYBQ-4 on thigh infections induced by MRSA USA300 and MSSA 29213 in mice (6-week-old female C57BL/6J mice) were evaluated. According to a previous description (45), two consecutive intraperitoneal injections of cyclophosphamide 4 days (150 mg/kg body weight) and 1 day (100 mg/kg body weight) before infection resulted in severe neutropenia (in 100 patients; neutrophils/mm^3^). Mice were infected by intramuscular injection of 0.1 mL of S. aureus culture (5 × 10^5^ CFU) into the right thigh. At 2 h postinfection, mice (*n* = 4) were treated with WYBQ-4 at single subcutaneous doses of 90 and 45 mg/kg, while mice treated with ceftaroline fosamil at the same doses served as positive controls. Then, 24 h after infection, mice were euthanized, and the right thighs were aseptically removed, weighed, homogenized, and applied to nonresistant BHI plates for CFU counting.

### Mouse systemic infection model.

The S. aureus-induced systemic infection model of mice (6-week-old female C57BL/6J mice) was established as previously described (46). The mice (6 per group) were inoculated with MRSA USA300, clinical isolate SA28, or SA1B2B (5 × 10^7^ CFU per mouse) by intraperitoneal injection and treated with different doses of WYBQ-4, cefepime, or moxalactam at 0 and 3 h postinfection by tail intravenous injection. The mice infected in the same way were treated with physiological saline as an infection control group. The mock group was not infected with S. aureus, and the 7-day mortality of the mice in each group was calculated.

### Statistical analysis.

All data are presented as the mean ± standard deviation (SD). Statistical analyses were performed using Prism software v8.0 (GraphPad Software, USA). Student’s *t* tests (two-tailed) were used to compare two data sets. The log-rank test was used for survival analysis. *P < *0.05 was considered statistically significant.

### Ethical statement.

The animal experiments were conducted in accordance with the principles of the Basel Declaration and the guidelines of the Animal Care and Use Committee of Jilin University; the IACUC (Institutional Animal Care and Use Committee) number is 201904017.

### Data availability.

All data generated during this study are available upon request from the corresponding authors.
