# Supplementary material for: WYBQ-4: a New Bactericidal Agent against Methicillin-Resistant Staphylococcus aureus
Source: Microbiol Spectr. 2022 Sep 13;10(5):e00547-22. doi: 10.1128/spectrum.00547-22 (PMC9603521; doi:10.1128/spectrum.00547-22)
Supplement: Supplemental file 1 — Supplemental text, Table S1, and Fig. S1 and S2. Download spectrum.00547-22-s0001.pdf, PDF file, 0.3 MB [file spectrum.00547-22-s0001.pdf]

Supplementary Information to

WYBQ-4: a new bacteriostatic agent against methicillin-resistant *Staphylococcus aureus*

Shuhan Guan[1,2 †], Hangqian Yu[1 †], Hua Xiang[3], Li Wang[1,4], Jingyu Liu[1], Anfang Wu[1], Jianze Zheng[1], Hongbo Dong[5 *], Lin Wang[2 *], Dacheng Wang[1 *]

[1] College of Animal Science, Jilin University, Changchun 130062, China.

[2] State Key Laboratory for Zoonotic Diseases/Institute of Zoonosis/College of Veterinary Medicine, Jilin University, Changchun, China.

[3] College of Animal Medicine, Jilin Agricultural University, Changchun 130118, China.

[4] Changchun University of Chinese Medicine, Changchun, China.

[5] School of Pharmacy, Chengdu University, Chengdu 610052, China.

* Corresponding author: Dacheng Wang, wangdc@jlu.edu.cn; Lin Wang, wanglin1020@jlu.edu.cn; Hongbo Dong, donghongbo@cdu.edu.cn

†These authors contributed equally.

Contents

Supplementary Method. 1 Determination of the kinetic parameters for interactions of WYBQ-4 with PBP2a.

Supplementary Table. 1 Strains of bacteria used in this study.

Supplementary Figure. 1 The ^{1}H NMR data of WYBQ-4.

Supplementary Figure. 2 The HRMS (ESI) data of WYBQ-4.

Supplementary Method. 1 Determination of the Kinetic Parameters for Interactions of WYBQ-4 with PBP2a.

Determination of the kinetic parameters for interactions of WYBQ-4 with PBP2a

The kinetic parameters of the interaction of WYBQ-4 with PBP2a were determined as previously described (1). The kinetics of the reaction between PBP2a of MRSA with WYBQ-4 was studied in homogeneous aqueous solution at 37°C in 25 mM Hepes buffer, pH 7.0, containing 1 M NaCl and the inhibition of PBPs by β-lactams follows this kinetic scheme.

$$E + I \overset{ks}{\Leftrightarrow} EI \overset{k2}{\rightarrow} E - I \overset{k3}{\rightarrow} E + P$$

In this minimal scheme, E represents PBP2a, EI is the noncovalent preacylation complex, E-I is the covalent acyl-enzyme species, and P denotes the product of E-I hydrolysis and restoration of enzyme activity.

The typical reactions of β-lactam could be monitored through competition with a chromophoric β-lactam, nitrocefin, or directly in certain cases by fluorescence intensity of protein. In this study, it is proposed to determine the secondary rate constants (k_2/Ks) of WYBQ-4 by nitrocefin competition assay. The reaction was conducted in the above buffer, and the reaction system contained PBP2a (2.5 μM) and different concentrations of nitrocefin (20-120 μM). The reaction was carried out in a UV spectrophotometer (PERSEE T600, China) and the change in absorbance at 500 nm was monitored for 45 min.

Apparent first-order rate constants for acylation by nitrocefin were determined by fitting the change in absorbance with time to eqn. (1):

$$A = A_0 + \Delta\varepsilon_n E_0[(1 - \exp(-k_n^* t)] + k_0 N_0 \Delta\varepsilon_n E_0 t \quad (1)$$

where A_0 is the initial absorbance at 500 nm, Δεn the change in molar absorption coefficient on nitrocefin hydrolysis (15900 $M^{-1}cm^{-1}$), E_0 the initial enzyme concentration, N_0 the initial nitrocefin concentration, kn* the (burst) pseudo-first-order rate constant for acylation of PBP2a by nitrocefin, and k_0 the (line) second-order rate constant of a non-specific hydrolysis of nitrocefin in the presence of PBP2a. The second-order rate constants (kn) for acylation were calculated by fitting a plot of the apparent first-order rate constants (kn*) against the concentration of nitrocefin using a linear least-squares method.

Subsequently, nitrocefin (100 μM) was used as a reporter molecule to determine the apparent first-order rate constants for acylation of WYBQ-4 at different concentrations (0-500 μM) in competition assays. In this case, the change in absorbance at 500 nm with time was fitted to eqn. (2):

$$A = A_0 + \Delta\varepsilon_n E_0 \left(k_n^* / (k_n^* + k_i^*)(1 - exp\left(-(k_n^* + k_i^*)t\right)\right) + k_0 N_0 \Delta\varepsilon_n E_0\, t \quad (2)$$

Where kn* is the pseudo-first-order rate constant for acylation by nitrocefin at the concentration (N_0) employed (0.1 mM), ki* the pseudo-first-order rate constant for the reaction of PBP2a with the WYBQ-4, E_0 the initial enzyme concentration (2.5 μM), and k_0 the second-order rate constant of nonspecific nitrocefin hydrolysis. Second-order rate constants (ki) for acylation were calculated from a linear least-squares fit to a plot of the apparent first-order rate constants (ki*) against the concentration of WYBQ-4. All plots were linear for the concentrations of WYBQ-4 used.

Determination of dissociation equilibrium constants (Ks) of WYBQ-4 and PBP2a

The dissociation equilibrium constant (*Ks*) of PBP2a with WYBQ-4 were determined by the BOCILLIN method (2), as previously described. First, the standard curve of BOCILLIN FL with PBP2a was established. The reaction system contained BOCILLIN FL (10 μM) and different final concentrations of PBP2a protein (0-40 μM). After mixing for 20 min, 15 μL of sodium dodecyl sulfate sample buffer was added and boiled for 3 min. Samples (30 μL) were loaded onto a 10% SDS-polyacrylamide gel, which was developed and then observed using a fluorescence imager to calculate the grayscale curve. Subsequently, 15 μM of PBP2a andWYBQ-4 (0-90 μM) were reacted in a reaction 60 μL system for 45 min, and BOCILLIN FL was added to detect the free protein when the reaction reached equilibrium to obtain the *Ks*.

The deacylation rate constants for wild-type PBP2a were determined using BOCILLIN FL as a reporter molecule. The reaction mixture (60 μL) contained 15 μM of PBP2a and WYBQ-4 concentration greater than 2 times higher than the *Ks* value. The mixture was incubated in 25 mM Hepes, 1 M NaCl (pH 7.0) buffer for 45 min at room temperature. A tiny molecular sieve was used to filter out extra WYBQ-4.

The mixture was diluted 5 times with buffer and incubated at room temperature for different time intervals. The amount of the free protein, liberated from the acyl protein species, was assayed by the addition of BOCILLIN FL to afford a final concentration of 10 μM and incubated for an additional 20 min at room temperature. After 10% polyacrylamide gel electrophoresis and observation under a fluorescent gel imager (Azure c600, USA). Grayscale analysis was performed by imageJ and the rate constant *k3* for the deacylation reaction of WYBQ-4 with PBP2a was determined based on eqn (3):

$$\ln\frac{PBPt}{PBP0} = -k_3 t$$

PBPt is the residual acylase concentration at time t and *PBP0* is the initial concentration of the acylase complex.

Supplementary Table.1 Strains of bacteria used in this study

Strains	Source/Reference
**Gram-positive bacteria**	
MRSE	ATCC 12228
MSSE	ATCC 35984
MRSA	ATCC BAA-1717 (USA 300)
MRSA COL	In this study
MSSA	Newman
MSSA	ATCC 29213
Enterococcus faecium	ATCC 700221
Enterococcus faecalis	ATCC 29212
Bacillus subtilis PY79	BioVector NTCC
**Gram-negative bacteria**	
Escherichia coli	ATCC 25922
Escherichia coli	ATCC 35218
Escherichia coli DH5a	Invitrogen
Klebsiella pneumoniae	ATCC 700603
Pseudomonas aeruginosa	ATCC27853
Acinetobacter baumannii	In this study
Enterobacter aerogenes	In this study
Streptococcus pyogenes	ATCC 19615
Enterobacter cloacae	In this study
**Plasmids**	
pET-28a	Miaolingbio

ATCC, American Type Culture Collection; MRSA, methicillin-resistant *Staphylococcus aureus*; MSSA, methicillin-sensitive *Staphylococcus aureus*; MRSE, methicillin-resistant *Staphylococcus epidermidis*; MSSE, methicillin-sensitive *Staphylococcu*s epidermidis.

Supplementary Figure. 1 The ^{1}H NMR data of WYBQ-4.

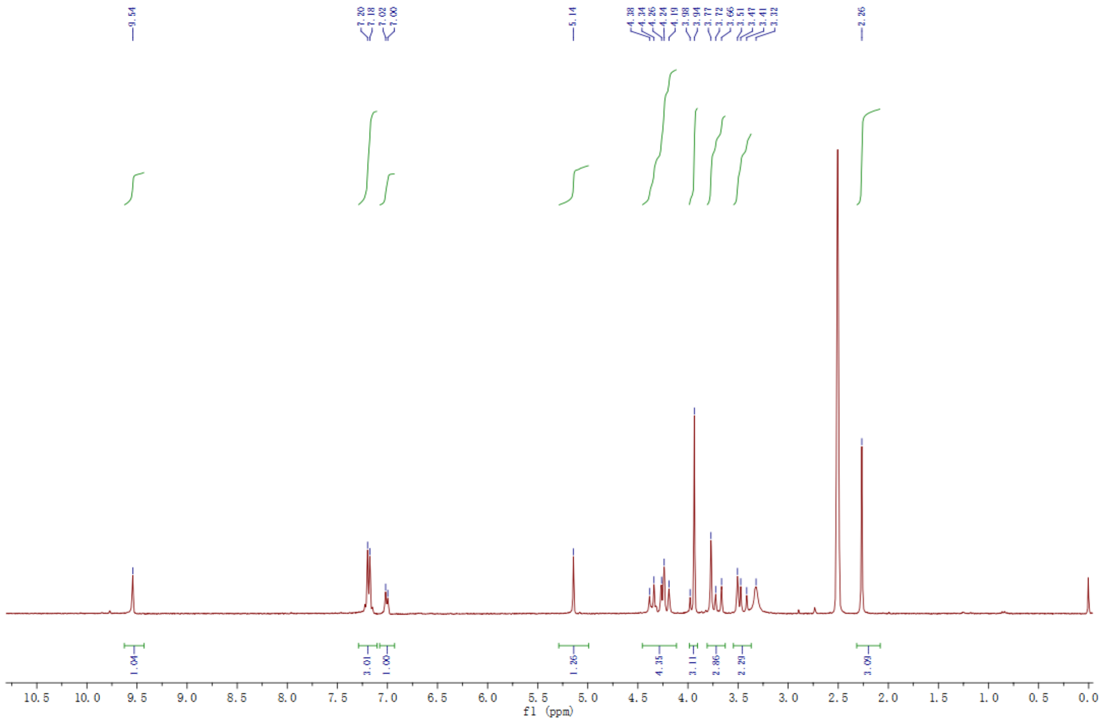

^{1}H NMR (DMSO-d_6, 400 MHz) δ 9.54 (s, 1H, -NH), 7.20-7.18 (m, 3H, -Ph), 7.02-7.00 (m, 1H, -Ph), 5.14 (s, 1H, -NCHS-), 4.38-4.19 (m, 4H, -OCH_2-, -SCH_2-), 3.94 (s, 3H, N-CH_3), 3.77-3.66 (m, 2H, -SCH_2-), 3.51-3.41 (m, -SCH_2-), 2.26 (s, 3H, -$PhCH_3$).

Supplementary Figure. 2 The HRMS (ESI) data of WYBQ-4.

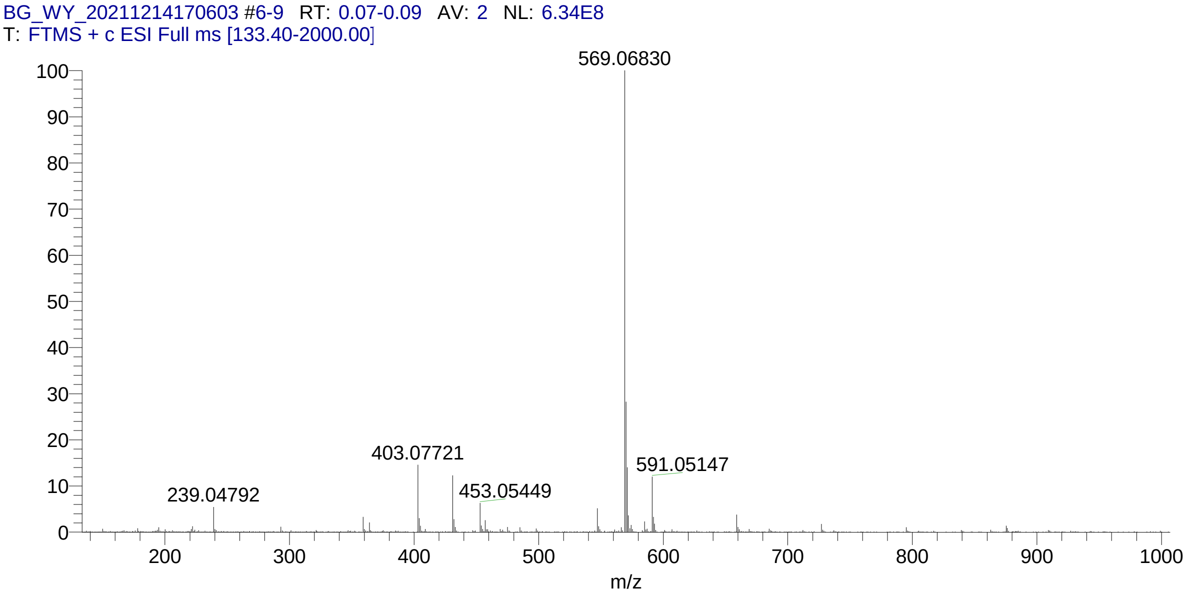

HRMS (ESI) m/z $[M+Na]^+$ calcd for $C_{22}H_{22}N_6O_5S_3Na^+$ 569.0706, found 569.0683.

References

1. Graves-Woodward K, Pratt RF. 1998. Reaction of soluble penicillin-binding protein 2a of methicillin-resistant Staphylococcus aureus with beta-lactams and acyclic substrates: kinetics in homogeneous solution. Biochem J 332 (Pt 3):755-61.
2. Pal S, Ghosh AS. 2019. PBP Isolation and DD-Carboxypeptidase Assay. Methods Mol Biol 1946:207-225.
